# Balancing risks and benefits: clinicians’ perspectives on the use of generative AI chatbots in mental healthcare

**DOI:** 10.3389/fdgth.2025.1606291

**Published:** 2025-05-29

**Authors:** Lyndsey Hipgrave, Jessie Goldie, Simon Dennis, Amanda Coleman

**Affiliations:** ^1^ Melbourne School of Psychological Sciences, The University of Melbourne, Parkville, VIC, Australia; ^2^ Brain and Mental Health Hub, The University of Melbourne, Parkville, VIC, Australia; ^3^ School of Psychological Sciences, Monash University, Clayton, VIC, Australia

**Keywords:** generative AI, chatbots, mental health, mental health clinicians, benefits risks

## Abstract

**Introduction:**

The use of generative-AI chatbots has proliferated in mental health, to support both clients and clinicians across a range of uses. This paper aimed to explore the perspectives of mental health clinicians regarding the risks and benefits of integrating generative-AI chatbots into the mental health landscape.

**Methods:**

Twenty-three clinicians participated in a 45-minute virtual interview, in which a series of open-ended and scale-based questions were asked, and a demonstration of a mental health chatbot's potential capabilities was presented.

**Results:**

Participants highlighted several benefits of chatbots, such as their ability to administer homework tasks, provide multilingual support, enhance accessibility and affordability of mental healthcare, offer access to up-to-date research, and increase engagement in some client groups. However, they also identified risks, including the lack of regulation, data and privacy concerns, chatbots' limited understanding of client backgrounds, potential for client over-reliance on chatbots, incorrect treatment recommendations, and the inability to detect subtle communication cues, such as tone and eye contact. There was no significant finding to suggest that participants viewed either the risks or benefits as outweighing the other. Moreover, a demonstration of potential chatbot capabilities was not found to influence whether participants favoured the risks or benefits of chatbots.

**Discussion:**

Qualitative responses revealed that the balance of risks and benefits is highly contextual, varying based on the use case and the population group being served. This study contributes important insights from critical stakeholders for chatbot developers to consider in future iterations of AI tools for mental health.

## 1 Introduction

The adoption of artificial intelligence (AI) in mental healthcare presents an array of possible opportunities and risks. AI chatbots have been studied to support a range of tasks benefiting clinicians and patients, including therapy (1, 2), administrative tasks (3, 4), patient screening (5, 6), diagnosis (7), and psychoeducation and training (8, 9). Literature suggests benefits for AI chatbots in mental healthcare including improvements in mental distress (10), affordability of care (11), 24/7 availability (2), multilingual support (12), streamlining record-keeping and data organisation (13), early intervention or prevention (14–16), delivery of care in an empathetic manner (8, 10), and reduced stigma in help-seeking (17, 18). Generative AI, powered by natural language processing, offers unique advantages over its non-generative AI predecessors, including advanced natural language understanding that enables more empathetic, human-like, and engaging interactions, enhancing patient comfort and overall engagement (19–21). It maintains contextual awareness over long conversations (22) and can personalise responses based on previous interactions. Its ability to continuously improve and learn from new data allows for ongoing enhancement of responses and capabilities (22). It effectively handles complex, open-ended queries and can offer detailed information which could support diagnostic and treatment decision-making tasks (23). These unique capabilities of generative AI highlight its potential to revolutionise mental health support.

However, challenges have been identified regarding the use of generative AI in mental health (24, 25). Many research articles highlight concerns around security, privacy, and confidentiality (26–28). Other concerns include AI's handling of disclosures of criminal activity (29), the lack of comprehensive regulatory frameworks (30, 31), chatbots' inabilities to fully grasp the complexities of individual client situations (32, 33), and the risk of AI suggesting inappropriate diagnoses or treatment recommendations (34, 35). The unpredictability of generative AI chatbots, which generate new responses each time, poses risks of producing inappropriate or harmful replies (34). It also risks generating “hallucinations,” where the AI generates information that is incorrect, misleading, or entirely fabricated (25, 36). Additionally, generative AI can inadvertently reproduce and amplify biases present in its training data (30, 37). Mitigating risks and ensuring safe, effective integration into mental healthcare requires careful consideration, collaboration, and robust regulatory frameworks.

While studies have begun examining the broader use of generative AI in mental health, there is limited research into mental health clinicians' perspectives on these tools (3, 38). This gap is concerning, given the potential impact of these tools on clinical workflows and patient care. Existing studies show varying levels of experience with chatbots, and a range of views on the benefits and risks of AI in mental healthcare (3, 38–42). For instance, a global survey of physicians' attitudes towards the integration of artificial intelligence in mental healthcare found that 40% were uncertain that the benefits of adopting AI into the profession would outweigh the risks, with the authors speculating that a lack of awareness and general scepticism may be contributing to the apprehension (38). Moreover, another study found that while adoption of chatbots was low amongst mental healthcare professionals, those who had used them reported mostly satisfactory experiences (39). Evaluating clinicians' insights into the risks and benefits of chatbots following a demonstration the technology's capabilities will provide essential information for optimising AI-driven interventions that are both effective and aligned with clinical practice standards. By observing a chatbot's performance across a range of use cases, participants will gain a tangible and standardised reference point from which to evaluate its functionality, limitations, and clinical relevance. This novel methodological approach will provide access to feedback grounded in observed capabilities rather than prior expectations or conjecture.

The primary aim of this study is to explore mental health clinicians' perceptions of using AI chatbots in mental health support, specifically focusing on their views regarding the risks and benefits. This study will first explore mental health clinicians' perceptions of the risks and benefits associated with using a generative-AI chatbot for mental health support. The study will also assess whether, from the perspective of clinicians, the perceived benefits of generative-AI chatbots outweigh the perceived risks, or vice versa. It is expected that clinicians will perceive the risks of using AI chatbots in mental health support to outweigh the benefits. Finally, the study will investigate the extent to which a generative-AI chatbot demonstration influences clinicians' views on its risks and benefits. It is anticipated that following exposure to the chatbot's capabilities, clinicians' views will shift in favour of the benefits, reflecting a more positive perception of AI chatbots.

## 2 Method

### 2.1 Participants

A range of mental health professionals were recruited through convenience sampling due to the exploratory nature of the study. A study flyer was distributed across relevant professional and personal networks, including university mailing lists, online discussion groups and member-only forums for mental health professionals, social media platforms (e.g., LinkedIn, Facebook), and via word-of-mouth referrals from professional contacts. Participants were invited to take part in a 45-min interview over Zoom. Recruitment continued until the pre-agreed deadline, by which point no additional participants had expressed interest. While convenience sampling carries the risk of selection bias, and data saturation was not assessed through thematic redundancy, the sample reflected diversity in profession, experience level, and age. Moreover, the range of perspectives expressed, both supportive and critical, suggests that the sample was not disproportionately skewed toward any single viewpoint regarding AI chatbots. In total, 23 participants were recruited and interviewed for the study. Participant characteristics are presented in Table 1.

**Table 1:** Demographic characteristics of the study sample.

Demographic	*n* (%)	*M (SD)*
Gender		
Female	17 (73.9)	
Male	6 (26.1)	
Nationality		
Australian	19 (82.6)	
Norwegian	1 (4.3)	
Irish	1 (4.3)	
Uzbekistani	1 (4.3)	
Filipino	1 (4.3)	
Profession		
Counsellor	5 (21.7)	
Mental health support worker	3 (13.0)	
Provisional psychologist	6 (26.1)	
Social worker	3 (13.0)	
Psychologist	4 (17.4)	
Psychiatrist	2 (8.7)	
Years of professional experience		10.8 (14.3)
Age		39.4 (16.2)

*N* = 23. Percentages may not add up to 100 based on rounding.

### 2.2 Materials

#### 2.2.1 Questionnaire

To address all three research aims, a single questionnaire was presented to participants who consented to the study. The questionnaire began with the collection of demographic information, followed by 13 questions that included both structured interview items and scale-based questions (the full questionnaire is available in Supplementary Material A). The first two questions explored clinicians' levels of experience with chatbots and understanding of mental health chatbots. Questions 3 and 4 were open-ended, inviting participants to share their views on the perceived benefits and concerns of using an AI chatbot for mental health support. Questions 5 and 6 used a Likert scale of 1 (strongly disagree) to 4 (strongly agree) to assess participants' perspectives on the balance of risks vs. benefits and their likelihood of recommending a chatbot for mental health support. Question 7 asked participants to select and explain their top three preferences from a list of potential uses of chatbots in mental health. In question 8, participants were asked to rate their agreement with 18 risk-benefit statements on a scale of 1 (strongly disagree) to 4 (strongly agree). Question 9 sought clinicians' perspectives on the ability for chatbots to adequately manage crises including suicidal intentions or admission of illegal activity. Following question 9, an 11-minute, silent demonstration video was played to participants. Once the clip was played, some follow-up questions were asked to assess the impact of the demonstration. This included repeating the scale-based questions (5 and 6), a question about how the demonstration affected participants' understanding of chatbots, and an invitation to provide general comments or feedback.

#### 2.2.2 Chatbot demonstration

The demonstration featured a screen-recorded video of a series of GPTs created using ChatGPT 4o. The demonstration followed a hypothetical scenario in which Saman, a recent immigrant to Australia from Sri Lanka, was seeking help for suspected depression following a challenging transition to Australia. Using a chatbot, Saman was taken through triage and assessment, onboarding, counselling and a gratitude exercise. The last demonstration showed Saman's therapist using the chatbot to organise and summarise their case notes, search and synthesize literature relevant to Saman's situation, and suggest future interventions and a relevant worksheet to give to Saman. The chatbot transcript is provided in Supplementary Material B.

### 2.3 Procedure

Ethics approval was obtained from the Office of Research Ethics and Integrity at the University of Melbourne. Once participants registered their interest and were deemed eligible, a consent form and plain language statement was sent for them to complete, along with a link to select an interview time slot. Interviews were conducted by either or both of the first two authors. During the interview, questions were read aloud, and for those with multiple items or selection options, screensharing was used to allow participants to read the statements themselves. The demonstration was also presented through screensharing.

## 3 Results

A combination of quantitative and qualitative data were collected. The specific quantitative analyses for each research question are detailed under their respective headings. The qualitative data were transcribed using AI transcription service Notta, after which a qualitative thematic analysis was performed using Braun and Clarke's (2006) six-phase approach to identifying, analysing, and reporting patterns (themes) within the data. Manual coding was performed given the manageable sample size, which allowed for an in-depth and thorough examination of each interview. Two coders, the first and second authors, independently reviewed transcripts. Codes were developed iteratively using a data-driven (inductive) approach and discussed in consensus meetings. Inter-coder agreement was established through regular discussion and revision.

### 3.1 Clinicians' perceptions of the risks and benefits of generative-AI chatbots

Participants were asked to rate their agreement with 18 risk-benefit statements on a scale of 1 (strongly disagree) to 4 (strongly agree) to uncover their views of the risks and benefits of integrating AI into mental healthcare. Although Likert-type items are ordinal in nature, we treated them as approximately interval-level data to enable parametric analyses. Each item was analysed separately rather than combined into a composite score, however the statements were developed as part of a structured and conceptually coherent questionnaire. As the study relied on single-item measures, we adopted this approach cautiously and acknowledge the limitations of treating ordinal data as approximately interval-level. Nonetheless, this analytic strategy is commonly used in psychological research (43) and was deemed appropriate given the structured design of the item set and the exploratory aims of the study. Descriptive statistics and a one-sample *t*-test were analysed to determine whether the mean significantly deviated from the middle value of 2.5. Results provided in Table 2 provide insight into mental health clinicians' perceptions of the risks and benefits of utilising generative-AI chatbots for mental health support. Participants' views significantly deviated from neutrality on many of the statements, suggesting strong views on topics broached in the questionnaire.

**Table 2:** Summary of descriptive statistics and one-sample *t*-test for 18 items.

Item	*n*	*M (SD)*	Median	Range	*t*	*df*	Cohen's *d*
AI chatbots reminding clients of homework will be helpful.	23	3.87 (0.34)	3	1–4	19.07***	22	3.98
Multilingual AI chatbots will be beneficial.	23	3.87 (0.34)	3	1–4	19.07***	22	3.98
24/7 AI chatbots will benefit clients.	23	3.70 (0.56)	2.5	1–4	10.26***	22	2.14
AI chatbots updated with research will be beneficial.	23	3.70 (0.56)	4	2–4	10.26***	22	2.14
Lack of regulation for AI care poses risks.	23	3.61 (0.66)	4	2–4	8.10***	22	1.69
AI chatbots pose security and privacy risks.	22	3.50 (0.74)	4	3–4	6.34***	21	1.35
AI chatbots will make mental health support accessible and affordable.	23	3.35 (0.78)	3	1–4	5.25***	22	1.09
Non-judgmental chatbots will increase client engagement.	22	3.32 (0.89)	4	3–4	4.29***	21	0.92
AI chatbots will lack understanding of clients’ backgrounds.	23	3.17 (0.98)	4	1–4	3.28**	22	0.69
AI chatbots responding to social media could benefit clients.	22	3.14 (0.71)	3	2–4	4.20***	21	0.90
AI chatbots will support early intervention and prevention.	22	3.05 (0.84)	3	1–4	3.03**	21	0.65
Clients may over-rely on AI chatbots for support.	22	3.00 (1.02)	3	1–4	2.29*	21	0.49
AI chatbots may give incorrect treatment recommendations.	22	2.96 (0.58)	3	2–4	3.71***	21	0.79
AI chatbots will lack empathy and connection with clients.	23	2.91 (1.00)	4	1–4	1.99	22	0.42
AI chatbots may misdiagnose client issues.	22	2.82 (0.80)	3	2–4	1.88	21	0.40
AI chatbots will avoid bias and stereotyping.	22	2.55 (0.96)	2	1–4	0.22	21	0.05
AI chatbots offering intensive therapy between sessions is beneficial.	23	2.13 (1.06)	2	1–4	−1.68	22	−0.35
AI chatbots may suggest harmful or illegal activities.	20	1.80 (0.77)	3.5	1–4	−4.08***	19	−0.91

Items have been abbreviated. Items appear in descending order of mean result. See Supplementary Material A for original order of items. *p < .05, **p < .01, ***p < .001. For the Student's *t*-test, the alternative hypothesis specifies that the mean is different from 2.5 (the middle value).

#### 3.1.1 Benefits

Clinicians recognised several advantages of AI chatbots in mental healthcare. The strongest agreement was for a chatbot's ability to remind clients of routine, low-risk homework activities, and for the bot to provide multilingual support, both with a mean response of 3.87 (*SD* = 0.34). The 24/7 availability of AI chatbots was also highly rated (*M* = 3.70, *SD* = 0.56), supporting the observation that clinicians view improvements in accessibility as being a major benefit of chatbots. One participant added that the availability of chatbots would “*widen the funnel of the amount of people that can get into therapy [and] … free up more space and time for therapists to see more high needs complex cases”* (#3, M, 26). Clinicians also believed that AI chatbots updated with the latest research would be beneficial (*M* = 3.70, *SD* = 0.56). One participant who worked as an National Disability Insurance Scheme (NDIS) employee noted the benefit of turning to ChatGPT for suggestions amidst an under resourced workforce, suggesting a level of trust exists in the advice provided by the bot:

“I find that they [employees] are usually overworked. So you feel kind of like you're bothering them [supervisors] a little bit if you're asking about certain things […] it would be good to just check with ChatGPT to kind of get some reassurance or some ideas in how to handle a specific situation” (#2, F, 27).

In contrast, another participant, a provisional psychologist, expressed concern around prospective clinicians with large University debts using chatbots to “*cut[ting] corners to save money and using something like a bot to get supervision rather than spending the money that they should be on a real supervisor who's going to give them like their 15, 20, 30 years of knowledge”* (#7, F, 27).

Participants agreed with the statement that AI chatbots would improve accessibility and affordability of mental health support (*M* = 3.55, *SD* = 0.78). The qualitative analysis revealed some nuance to this view. One participant described the potential advantage for a particular population: “*It's accessible for a lot of people, a lot more people. Psychs are obviously not overly accessible and obviously driven on wait periods a lot of the time especially in rural and regional communities”* (#15, M, 26), while another similarly noted benefits for young people, who “*are so much more used to just texting or emailing or seeing written responses rather than calling or audio note or face to face”* (#7, F, 27). Others expressed concerns around accessibility for other demographics, such *as “older generations who don't have access to an iPhone or a computer”* (#1, F, 25) or for clients facing “*trauma, social disadvantage, [and] homelessness”* (#8, F, 58).

Participants agreed that if bots were perceived to be non-judgemental by some clients, that this could increase engagement (*M* = 3.52, SD = 0.89). One participant who worked with autistic children described a case in which one of his clients, an autistic boy with social anxiety, was able to better engage by using a chatbot tool collaboratively in the therapy room: “*I was watching him like blossom compared to the way he usually is […] It's like it just provides you that lower intensity social experience that you might be able to use to build up”* (#4, M, 42). Lastly, some clinicians also suggested that chatbots could play a role in early intervention and prevention (*M* = 3.05, *SD* = 0.84), and that monitoring social media activity could benefit certain clients (*M* = 3.14, *SD* = 0.71).

#### 3.1.2 Risks

Clinicians viewed the lack of regulation in AI chatbot usage (*M* = 3.61, SD = 0.66) as the greatest concern. One participant expressed that regulation and oversight of these tools would enable organisations to be held accountable for high-risk situations:

“There needs to be […] an organisation that's that actually holds accountability ethically for what occurs. Because even just then in that video, when the person said, I feel suicidal, who gets flagged to follow up on that?” (#22, F, 26)

Data security and privacy risks (*M* = 3.50, SD = 0.74) were also flagged as a concern, indicating a fear that AI chatbots may mishandle sensitive client information. Participants generally agreed that AI chatbots may lack sufficient understanding of clients' backgrounds (*M* = 3.17, SD = 0.98), suggesting that clinicians may view themselves as better positioned to manage ongoing relationships with clients with unique cultural, historical, and situational circumstances. Additionally, participants agreed that clients may over-rely on AI chatbots for support (*M* = 3.00, SD = 1.02), and that they may give inappropriate treatment recommendations (*M* = 2.96, SD = 0.58). One participant queried the treatment approach, specifically regarding the chatbot's choice of therapeutic orientation: “*I'm not sure what AI is disclosing about itself to sort of further the relationship. How to determine what school of therapy, I mean, as you know, the CBT, ACT, psychodynamic, schema therapy. So again, how on earth does AI decide?”* (#13, M 71).

A concern which emerged frequently across the qualitative responses was the inability for an AI chatbot to pick up on subtle details in human communication, particularly those only observable in a voice-to-voice or face-to-face context, such as physical presentation, eye-contact, speech, or tone. Clinicians viewed this shortcoming as posing a potential risk to the safety of the client through missed opportunities to identify and respond to escalating levels of distress, or to build a therapeutic relationship, which was expressed by many participants as being fundamental to the attainment of successful therapeutic outcomes. As one participant said,

“It might not be able to understand some of the very kind of nuances of human kind of emotions […] Sometimes we get patients who come in for about 10 sessions, they just say, I'm fine, I'm fine. There's nothing wrong with me. And it takes about 10 sessions to actually get to know what is the problem. So I don't know how much AI could deal with that in those kinds of situations when the patient says, I'm fine, when actually they're not” (#23, F, 47).

Interestingly, there was significant disagreement with the statement that AI chatbots would suggest harmful or illegal activities to clients (*M* = 1.80, *SD* = 0.77), suggesting that clinicians feel somewhat confident about some of the safety mechanisms embedded within chatbots.

### 3.2 Clinicians' views on whether benefits of generative AI chatbots outweigh the risks

To assess whether participants believed that the benefits of using AI technology in a therapeutic setting outweighed the risks, or vice versa, a one sample *t*-test was conducted. While participants were asked both pre- and post-demonstration about their views on whether risks or benefits outweighed the other, it was decided that the analysis would be undertaken on the post-demonstration data (see question 11 in Supplementary Material A), as it provides a more accurate reflection of how perceptions are likely to evolve with increased exposure to AI technologies. The mean response of 2.46 (*SD* = 1.06) was non-significant, *t*(21) = −0.20, *p* = .84, 95% CI [1.99, 2.92], revealing that overall clinicians' perceptions were largely neutral. There was a considerable amount of variance in the ratings, indicated by the standard deviation.

Participants' qualitative responses revealed a similar division. Several clinicians cited inexperience with the technology as a key factor in their uncertainty. Many participants had yet to engage with generative AI tools, with some citing rule-based chatbots, such as those used for online customer service purposes, as their primary experience with chatbots. Such customer service chatbots typically offer scripted responses based on keywords or specific user queries. Of those who had used generative AI, ChatGPT was the primary tool of use. While a small number of participants knew of the existence of mental-health-specific generative-AI chatbots, only one participant knew one by name. As such, some clinicians felt that their limited exposure to AI chatbots hindered their ability to form a confident judgment.

Importantly, many clinicians noted that the perceived benefits or risks of AI chatbots would be highly context-dependent, with the population group and specific purpose of the chatbot determining their views on the risks vs. benefits. For instance, participants expressed that the benefits of chatbots could outweigh the risks if their use was directed at low-risk clients, or to support administrative tasks, or for clients to complete routine, non-risky tasks, such as onboarding or therapeutic worksheets allocated and overseen by a therapist. Conversely, for clients with complex mental health backgrounds including psychosis or mania, the use of chatbots could present unique risks. As one participant highlighted,

“[…] is it possible that despite our best efforts, that the model could end up endorsing risky things psychologically […] colluding with the client towards sort of fantastical ideas, such that rather than their therapy process bringing them into sort of ever greater contact with the reality that they're living, it sort of ends up taking them away from that reality” (#4, M, 42).

Likewise, for clients presenting with suicidal ideation, several participants expressed that AI chatbots would be beneficial only if the technology was linked with services rather than employed as a standalone tool, so that users in crisis could be swiftly connected to a human:

“I guess I would believe that it [the chatbot] would probably adequately respond to [suicidal ideation] but manage not so much […] it is very beneficial to actually connect to a person in a situation like that. And also, you know, if it's something like suicide or something, obviously need to be linked to actual clinics” (#2, F, 27).

While not considered the preferred method for counselling, some clinicians acknowledged that AI chatbots could be useful in the short-term for certain clients. These results suggest that clinicians position their views around risks and benefits largely based on who uses the chatbots and for what purpose.

### 3.3 Findings from the chatbot demonstration

To investigate whether a demonstration of an AI chatbot's capabilities influenced clinicians' ratings of whether the benefits of using AI technology outweighed the risks, a one-tailed, paired-samples *t*-test was conducted. This test compared the mean scores of participants' ratings before and after the demonstration to analyse any significant differences. Additionally, a Bayesian analysis, which are particularly useful in small samples, was performed to assess the strength of the evidence for the observed effect (44).

Table 3 displays clinicians' mean ratings before and after the AI chatbot demonstration, showing a slight decrease in agreement that AI benefits outweigh risks in therapy from pre-demonstration (*M* = 2.59) to post-demonstration (*M* = 2.46). There was a slight, non-significant decrease in the perception that the benefits of AI chatbots in mental health outweigh the risks pre- and post- demonstration, *t*(20) = −1.00, *p* = .84, 95% CI [−0.52, ∞]. Additionally, the Bayes Factor was 0.25, suggesting that the null hypothesis, indicating no significant change in perceptions, was four times more likely than the alternative hypothesis, which proposed that the demonstration would shift views. These results suggest that the demonstration did not significantly alter clinicians' perspectives on whether its benefits outweigh the risks.

**Table 3:** Descriptive statistics of clinicians’ ratings on whether AI benefits outweigh risks in therapy.

AI benefits > risks in therapy?	*M (SD)*	95% CI [LL-UL]
Pre-demonstration (T1)	2.59 (0.85)	[2.21–2.97]
Post-demonstration (T2)	2.46 (1.06)	[1.99–2.92]

CI, confidence interval; LL, lower limit; UL, upper limit.

Qualitatively, several participants highlighted that the demonstration provided them with a clearer understanding of the chatbot's capabilities. Many were particularly enthused by the bot's summarising of case notes, “*I would have loved to have had access to something like that all through my career. I think that would have been fantastic”* (#21, M, 72). However, for some participants, the demonstration reinforced existing concerns, particularly regarding the chatbot's response to suicide risk. Specifically, the chatbot's suggestion to contact Lifeline without offering further support or initiating contact with services raised concerns about its adequacy in managing crises. While some participants were pleasantly surprised by the chatbot's ability to engage in an empathetic manner, others felt that its approach to counselling was lacking. One participant noted that the chatbot appeared to adopt a medical model approach, “*It feels like a medical model to just go symptom like treatment. And I get that that's appropriate, especially if there's some immediate need. But I also think that there's probably some other background and present information that was missing”* (#5, F, 41).

## 4 Discussion

### 4.1 Main findings

**Figure 1:**
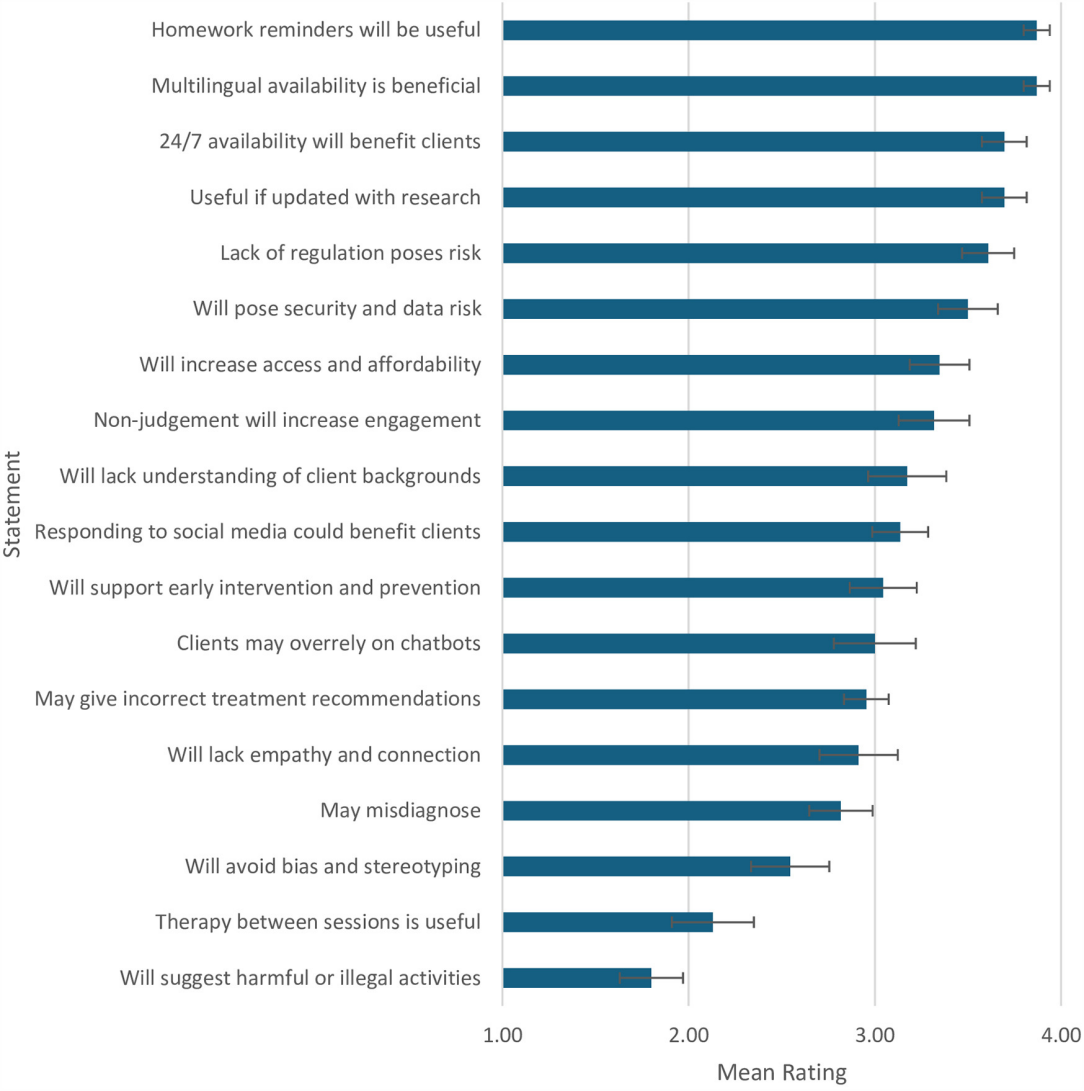
Mean ratings of participant agreement across risk/benefit statements*. *Items have been abbreviated. Items appear in descending order of mean result. See Supplementary Material A for original order of items.

This paper explored mental health clinicians' views on the risks and benefits of generative-AI chatbots in mental healthcare. Clinicians showed significant agreement with statements of the benefits of chatbots, including reminding clients of homework, offering multilingual support, 24/7 availability, providing evidence-based suggestions, improving accessibility, affordability, and client engagement through non-judgmental interactions, supporting early intervention, and monitoring social media for client wellbeing. Key risks included lack of regulation, data security and privacy concerns, limited understanding of clients' backgrounds, potential over-reliance on chatbots, and inaccurate treatment recommendations. Participants significantly disagreed with the notion that AI chatbots might suggest harmful or illegal activities (see Figure 1).

The qualitative findings in this study provide a level of depth not captured in previous research. While some participants were optimistic about chatbots offering evidence-based support, others were wary of AI being used to cut costs at the expense of professional oversight. Increased accessibility was seen as beneficial, though participants noted populations for whom chatbots may still be inaccessible. They also queried the way in which chatbots would decide on their treatment approach. Solutions were suggested in response to concerns about regulation, data privacy, and security, including embedding chatbots within services, ensuring human oversight, and limiting their use to non-critical tasks. Participants were also apprehensive about chatbots' limited ability to interpret the complexities of human communication. Though not named explicitly by participants, another important concern raised aligns with what has been described in AI ethics literature as *AI sycophancy*, which refers to the tendency of AI systems to prioritise user satisfaction over truthfulness or therapeutic challenge (45). In mental health contexts, this could lead to chatbots inadvertently reinforcing maladaptive beliefs, particularly among vulnerable users. This risk was reflected in one participant's concern that generative AI might collude with clients’ distortions of reality, rather than helping them move toward more accurate perspectives of their difficulties.

Contrary to expectations, the hypothesis that the risks of AI chatbots would significantly outweigh the benefits was not supported by the findings. The mean result of 2.46 reflects a level of uncertainty, further corroborated by the qualitative data. Although other studies also identified apprehensions (38, 40), our study provided additional qualitative insights. For some participants, limited experience was a factor; however, the wide range of potential applications also made it challenging to definitively favour either the risks or the benefits. Overall, participants were more supportive of the benefits of chatbots in low-risk contexts, particularly for administrative use cases and assisting clients with mild mental health challenges. However, they noted that for certain mental health conditions, chatbots could present additional safety risks. Echoing previous literature that responsible regulation is critical for the introduction of these tools into a delicate space (24, 46), participants expressed a preference for a collaborative approach, which would allow clinicians to intervene when necessary, ensuring that the limitations of AI at this nascent stage are managed and that client safety remains paramount.

Unlike earlier studies that suggested increased familiarity with AI tools would lead to greater endorsement of their use (39, 47), results in this study found that the chatbot demonstration did not significantly change clinicians' views on the balance of risks and benefits. The qualitative data provided further explanation for the results. While clinicians commented on preferred aspects of the chatbot after watching the demonstration, such as automating case notes, they also provided positive and negative feedback on the chatbot's communication capabilities. Some were impressed with the bot's natural, human-like communication skills, echoing previous literature (48), while others were concerned that not enough time was spent exploring the client's issues, which presented as a missed opportunity for connection and a dry approach to counselling. Moreover, some participants' concerns around chatbots' abilities to respond to crises were reinforced as a result of the bot's response to the client's disclosure of suicidal ideation.

Several limitations of the demonstration may help explain why clinician perceptions were not significantly altered. Without the ability to interact directly with the chatbot, clinicians may have found it difficult to conclusively assess how well it could navigate complex emotional content or tailor responses to client needs. The brevity of the demonstration further constrained participants' abilities to observe how the chatbot would build rapport, explore issues in depth, or manage an evolving therapeutic exchange. The demonstration did not address key systemic concerns raised by participants, such as regulation, data privacy, or the handling of confidential information. Additionally, some participants' past experiences with basic, rule-based chatbots may have hindered their appreciation of more advanced generative AI models. These factors point to the value of longer and more immersive approaches in future studies.

### 4.2 Implications

Consistent with previous research (3, 40, 42), this study highlights the need for more thorough training and exposure to AI chatbots for clinicians. Providing clinicians with opportunities for hands-on interaction with AI tools in real clinical settings, accompanied by robust training, will be crucial for helping them develop more informed and confident perspectives, and will enable field experts to be leading voices in the shaping of these tools.

This study takes place within a rapidly evolving field, where the capabilities of AI tools are advancing at an unprecedented pace. Many of the concerns raised by participants, such as the inability of chatbots to detect tone of voice or other subtle communication cues, are being actively addressed as technology continues to improve. For instance, ChatGPT's newly released voice capabilities allow for spoken interactions and better recognition of tone and context. These improvements help the chatbot respond more naturally and empathetically, addressing some of the key limitations raised. However, with these advancements comes a host of new risks, including but not limited to additional privacy concerns, misuse of sensitive data, or over-reliance on AI for complex mental health conditions. As such, it is crucial that ongoing research is conducted to keep pace with the rapid adaptation of these tools, and to ensure that they are safely and ethically integrated into mental healthcare.

One proposed pathway for safely integrating generative AI into mental healthcare is through a stepped-care model. Under this model, chatbots could support low-risk or routine tasks, such as psychoeducation, onboarding, or administrative support, while escalating higher-risk cases for human review. For example, triage systems could incorporate structured decision trees to identify markers of elevated risk, such as psychotic symptoms or disclosures of suicidal ideation, and initiate escalation protocols accordingly. Integration of such tools should be guided by best-practice suicide prevention and crisis response frameworks, including those published by organisations such as Australia's Beyond Blue or the United Kingdom's National Institute for Health and Care Excellence (NICE). By aligning chatbot functions with regulatory safeguards, stepped-care models offer a practical structure for balancing the benefits of AI-enhanced accessibility with the need to uphold client safety and ethical care.

In addition to aligning chatbot functions with stepped-care frameworks, future implementations must also comply with existing legal and ethical standards, for instance the General Data Protection Regulation (GDPR) in the European Union, the Health Insurance Portability and Accountability Act (HIPAA) in the United States, and relevant Australian privacy legislation (e.g., the Privacy Act 1988 and Australian Privacy Principles). These frameworks outline conditions for data collection, storage, and sharing, which should inform the development of AI chatbot infrastructure. Embedding such safeguards is critical to ensuring that AI-driven tools operate in ethically responsible and legally compliant ways, while maintaining public trust and client safety.

### 4.3 Limitations and future directions

This study had two major limitations. Firstly, the sample size was relatively small, potentially limiting statistical power and contributing to the lack of significant findings for the second and third research questions. Future studies would benefit from recruiting a larger, more diverse cohort to capture broader clinician perspectives. Secondly, many participants knew the interviewers directly or through mutual connections, possibly introducing demand characteristics, especially when providing feedback on the chatbot demonstration, which they knew was created by the interviewers. To minimise the effects of demand characteristics, it was not explicitly outlined to participants that the demonstration had been created by the interviewers. Rather, this information was only revealed if it emerged organically in conversation at the conclusion of the interview. Future research could use independent researchers to administer questionnaires, reducing potential bias. Another key direction is conducting trials where clinicians use chatbots over an extended period, offering a more accurate assessment of the benefits and limitations of these tools in clinical practice.

## 5 Conclusion

Generative AI chatbots hold potential to improve accessibility in mental healthcare, particularly with their 24/7 availability, affordability, remote accessibility, and multilingual capabilities. They also facilitate therapists' access to evidence-based research and provide more time to focus on complex cases. Participants recognised the benefits of chatbots in streamlining administrative tasks, such as client onboarding and case note documentation, and in supporting clients with routine tasks that do not require deep emotional engagement. However, clinicians will need education and training to build trust and familiarity with AI, allowing them to confidently incorporate these tools into their practice. To address some of the risks identified by participants, implementation of regulatory frameworks will be critical to ensure that users and their data remain safe, and stakeholders are held accountable for the responsible management of AI technologies in mental healthcare. Moreover, AI chatbots at their current stage of development are most likely to be beneficial when used as supplementary tools in a collaborative model, supporting routine tasks while allowing human therapists to focus on personalised, empathy-driven care that remains central to effective mental health treatment. Not all mental health treatment-seeking clients are suitable candidates for AI chatbot use and consequently, careful screening and oversight by clinicians is necessary to mitigate any further risks to clients' wellbeing. Overall, while clinicians have expressed optimism for the integration of AI tools into mental health, successful integration will require ongoing collaboration between developers, clinicians, researchers, and service users to ensure that AI tools are safe, ethical, and effective.

## Data Availability

The raw data supporting the conclusions of this article will be made available by the authors, without undue reservation.
